# Novel approaches for the rapid development of rationally designed arbovirus vaccines

**DOI:** 10.1016/j.onehlt.2023.100565

**Published:** 2023-05-13

**Authors:** Joyce W.M. van Bree, Imke Visser, Jo M. Duyvestyn, Muriel Aguilar-Bretones, Eleanor M. Marshall, Martijn J. van Hemert, Gorben P. Pijlman, Gijsbert P. van Nierop, Marjolein Kikkert, Barry H.G. Rockx, Pascal Miesen, Jelke J. Fros

**Affiliations:** a Laboratory of Virology, Wageningen University & Research, Wageningen, the Netherlands; b Department of Viroscience, Erasmus Medical Center, Rotterdam, the Netherlands; c Department of Medical Microbiology, Leiden University Medical Centre, Leiden, the Netherlands; d Department of Medical Microbiology, Radboud Institute for Molecular Life Sciences, Radboud University Medical Center, P.O. Box 9101, 6500, HB, Nijmegen, the Netherlands

**Keywords:** Live-attenuated vaccines, Arbovirus, Mosquito-borne virus, Flavivirus, Alphavirus, mosquito saliva, chimeric viruses, recoded viruses, pre-clinical model systems

## Abstract

Vector-borne diseases, including those transmitted by mosquitoes, account for more than 17% of infectious diseases worldwide. This number is expected to rise with an increased spread of vector mosquitoes and viruses due to climate change and man-made alterations to ecosystems. Among the most common, medically relevant mosquito-borne infections are those caused by arthropod-borne viruses (arboviruses), especially members of the genera *Flavivirus* and *Alphavirus*. Arbovirus infections can cause severe disease in humans, livestock and wildlife. Severe consequences from infections include congenital malformations as well as arthritogenic, haemorrhagic or neuroinvasive disease. Inactivated or live-attenuated vaccines (LAVs) are available for a small number of arboviruses; however there are no licensed vaccines for the majority of these infections. Here we discuss recent developments in pan-arbovirus LAV approaches, from site-directed attenuation strategies targeting conserved determinants of virulence to universal strategies that utilize genome-wide re-coding of viral genomes. In addition to these approaches, we discuss novel strategies targeting mosquito saliva proteins that play an important role in virus transmission and pathogenesis in vertebrate hosts.

For rapid pre-clinical evaluations of novel arbovirus vaccine candidates, representative *in vitro* and *in vivo* experimental systems are required to assess the desired specific immune responses. Here we discuss promising models to study attenuation of neuroinvasion, neurovirulence and virus transmission, as well as antibody induction and potential for cross-reactivity. Investigating broadly applicable vaccination strategies to target the direct interface of the vertebrate host, the mosquito vector and the viral pathogen is a prime example of a One Health strategy to tackle human and animal diseases.

## 1 Introduction

In recent years, the frequency, magnitude and global distributions of arthropod-borne (arbo)virus outbreaks have been fuelled by changes in climate, urbanization, human migration and population growth [[1], [2], [3], [4], [5], [6], [7]]. Increasing arbovirus prevalence can be ascribed to the expansion of mosquito vector populations, improved transmission efficiency and the adaptation of viruses to new host and vector species. Examples of important arbovirus (re-)emergence include the continuous global spread of dengue virus (DENV) and West Nile virus (WNV) and large outbreaks of chikungunya virus (CHIKV) and Zika virus (ZIKV) in the southern hemisphere [[8], [9], [10]]. Infection with these arboviruses can cause severe disease including congenital malformations as well as arthritogenic, haemorrhagic or neuroinvasive disease. Arboviruses may also infect livestock and wildlife, creating an animal reservoir that increases the chances of zoonosis exemplified by the spillover of WNV and Usutu virus (USUV) from the bird-mosquito transmission cycle to humans [11]. Most medically relevant arboviruses belong to the genera *Flavivirus* and *Alphavirus* and for most of these viruses no vaccines are available.

Licensed vaccines against yellow fever virus (YFV), Japanese encephalitis virus (JEV), dengue virus (DENV) and Venezuelan equine encephalitis virus (VEEV) for humans exist. Also for animals licensed vaccines against WNV, JEV and Getah virus exist. The first vaccine that protected from an arbovirus infection was the live-attenuated YFV strain 17D. YFV 17D originates from the Asibi isolate, isolated from an infected individual. This isolate was then passaged in monkeys and mice and finally over 200 times in chicken embryos (Reviewed in [12]). Due to its highly immunogenic character, inducing both innate and adaptive immunity that confer life-long protection, the live-attenuated YFV vaccine is considered one of the most successful human vaccines [13,14]. Although the vaccine is considered very safe, fatal adverse events can occur in immunocompromised individuals. Even though YFV 17D has been used for over 80 years, the molecular mechanisms for its attenuation remain poorly understood [15]. Recent studies found the genetic diversity of YFV 17D to be relatively limited compared to the originally isolated strain, and suggest narrow quasispecies diversity as a plausible correlate of attenuation [16]. The development of live-attenuated vaccines (LAVs) for other arboviruses has raised safety concerns because of the high mutation rate of viral RNA-dependent RNA polymerases [17]. High error rates may lead to mutations that can increase virulence of a vaccine strain, as observed for VEEV TC-83 and multiple CHIKV vaccine candidates [18,19]. Whole virus inactivated vaccines such as the licensed JEV vaccine IXIARO, are considered safer than LAV approaches. However, adjuvants and annual boosters are necessary for long-term protection [20].

The increasing frequency of arbovirus outbreaks and their societal impact stresses the need for reliable platforms that can aid in the rapid development of novel human and animal vaccines, broadly applicable for large groups of arboviruses [21,22]. One such infamous vaccine platform, due to the coronavirus pandemic, is the mRNA platform and variations thereof (e.g. self-amplifying mRNA vaccines). Multiple mRNA vaccine candidates have been developed for various arboviruses, including CHIKV, ZIKV and DENV (reviewed in [23]). Main advantages of mRNA vaccines are the short response time, scalability, reasonable production costs and safety profile. However, vaccine efficacy varies between different mRNA vaccine candidates and multiple boosters for longer duration of immunity are required [[24], [25], [26], [27], [28]]. LAVs, on the other hand, generally elicit a robust immune response with YFV strain 17D as a prime example. Therefore, safe-by-design attenuation strategies that prevent reversion to virulence are of considerable interest. Here we discuss potential approaches for the rational design of novel arbovirus LAVs, recent developments in pan arbovirus vaccine approaches and experimental models that are required for (pre-)clinical evaluations of arbovirus vaccine candidates.

## 2 Correlates of protection

Arboviruses enter vertebrate hosts when infected mosquitoes inject virus-containing saliva into the dermis during a blood meal. Detection of proteins from the mosquito saliva and viral particles by the host's pattern recognition receptors (PRRs), together with previous immune history and the host's genetic factors determine the nature of the acute, local phase of an arbovirus infection [[29], [30], [31]]. PRRs include membrane-bound Toll-like receptors 3, 7, and 8 [32,33], and cytosolic RIG-I-like receptors (RLRs) [32,[34], [35], [36], [37]], which activate signalling cascades that lead to the induction of type-I interferons (IFN) [[38], [39], [40], [41]]. The type-I IFN response, characterized by cytokine production and expression of IFN stimulated genes (ISGs), establishes a general antiviral state and is critical in controlling arbovirus infections. This is evident from the increased susceptibility of mice lacking the IFN receptor (*Ifnar1*^*−/−*^) to arbovirus infections [[42], [43], [44], [45], [46], [47]], compared to the protection offered by pre-treatment with IFN prior to infection [[48], [49], [50], [51]]. There is increasing evidence that the innate response can recollect previous infections via epigenetic reprogramming of innate immune cells to build a *de facto* innate immune memory called “trained immunity”, which comes into play during subsequent heterogenous and heterologous viral infections [52,53].

IFNs, cytokines and ISGs strengthen adaptive immune responses stimulating the generation of pathogen-specific active (CD4^+^ and CD8^+^) T-cells and B-cells. Both CD4^+^ and CD8^+^ T-cells play an important role in antiviral cytokine production and killing of infected cells. IFNs, cytokines and ISGs strengthen adaptive immune responses stimulating the generation of pathogen-specific active (CD4^+^ and CD8^+^) T-cells and B-cells. Both CD4^+^ and CD8^+^ T-cells play an important role in antiviral cytokine production and killing of infected cells. Knowledge on the precise immunologic responses to arbovirus antigens is still incomplete and many aspects (e.g. T-cell differentiation into the potential subtypes) are part of active investigations. CD8^+^ memory T-cells play a crucial role in the memory response as effective activation upon flavivirus re-infection generally prevents severe disease including central nervous system pathogenesis in neuroinvasive flavivirus infections [[54], [55], [56], [57], [58], [59], [60], [61]]. During alphavirus infection CD4^+^ T-cells supress viremia providing protection, while they also play a causative role in alphavirus pathogenesis [[62], [63], [64]]. For example, CHIKV infection or vaccination in mice elicits a CD4^+^ T-cell response which is associated with reduced viremia and increased joint pathology [65,66].

**Fig. 1:**
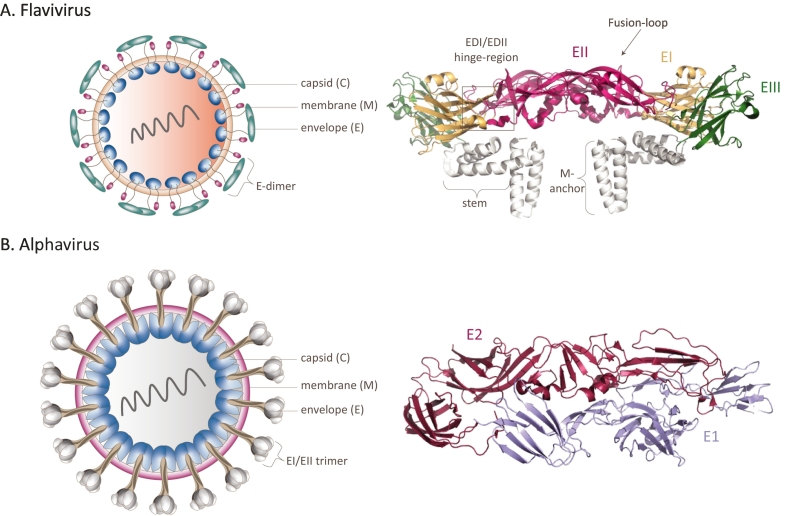
**Flavivirus and alphavirus particles.** Schematic representation of the spherical flavivirus (A) and alphavirus (B) particles (left). Capsid proteins (C) encapsidate the viral RNA surrounded by a lipid membrane. (A) The surface of the virion contains two proteins, the membrane protein (M) and envelope protein (E). The structure of aWest-Nile virus E homodimer is shown (A, right, PDB 7kva). Flavivirus E contains three domains (EDI-III); EDI is highlighted in yellow, EDII in magenta, and EDIII in green. Stem and M-anchor domains are coloured in white. (B) Alphavirus virions contain trimeric spikes of heterodimers that consist of viral envelope proteins E1 and E2 (left). The Chikungunya virus E1-E2 heterodimer structure is displayed (B, right, PDB 3n40). E2 and E1 proteins coloured in dark-red and lila, respectively. (For interpretation of the references to colour in this figure legend, the reader is referred to the web version of this article.)

Specialized CD4^+^ T-cells, called follicular helper T (TF_H_) cells, support the development of virus-specific B-cell memory responses and the production of high-affinity long-lasting antibodies after viral infections. The main targets for neutralizing antibodies against arboviruses are the envelope proteins (E). For flaviviruses, highly neutralizing antibodies targeting quaternary epitopes of E domain III (EDIII) and residues in the E domain I/II (EDI/EDII) hinge have been identified and isolated from animal models and humans (Fig. 1A) [[67], [68], [69]]. High titers of non-neutralizing antibodies are also produced upon flavivirus infection and generally target the fusion loop of E (FLE), pre-membrane (prM) and non-structural proteins (NS). In some cases there is evidence for neutralization-independent protection associated with these antibodies, e.g. antibody dependent cytotoxicity and antibody dependent complement deposition. Particularly, anti-flavivirus NS1 antibodies protect from infection and severe disease [[70], [71], [72], [73], [74], [75], [76]]. For alphaviruses, most potent neutralizing and protecting antibodies are generated against E1-E2 heterodimer glycoproteins (Fig. 1B). Antibodies against capsid (C) and non-structural proteins (nsP) upon alphavirus infection are also induced [[77], [78], [79]].

Of note, there is a high level of structural and sequence homology between different arbovirus species within the same genus, which can result in cross-reactive B- and T-cell responses [[80], [81], [82], [83], [84]]. For alphaviruses, neutralizing antibodies against conserved epitopes in E2 can provide cross-protection between closely related alphavirus species [85,86]. Moreover, poorly neutralizing antibodies raised against conserved epitopes in E1 were able to protect against more distantly related arthritogenic and encephalitic alphaviruses [87]. While homology between flavivirus EDIII is limited, EDII is highly conserved across the *Flavivirus* genus. Since antibody responses generated against EDII are mainly non-neutralizing, the overall neutralization potential of cross-reactive antibodies is poor [88]. Only early after infection or immunization has antibody cross-protection to heterologous but closely related viruses been observed. For example, cross-protection between DENV and ZIKV infections wanes fast [89]. Moreover, in some cases cross-reactive antibodies can also aid virus entry, resulting in enhanced replication and potentially enhanced disease; a phenomenon termed “antibody-dependent enhancement (ADE)”. ADE has been extensively described for infection of the four different DENV serotypes and is a known feature described in laboratory studies of many pathogenic enveloped viruses of humans and animals [90,91]. Pre-existing DENV immunity is additionally associated with transplacental transmission of ZIKV in experimental models [92,93]. *Vice versa*, ZIKV-specific antibodies may be involved in enhancement of DENV infection [94,95]. Multiple other *in vitro* and *in vivo* studies demonstrated ADE during infection with various other flaviviruses, e.g. YFV, WNV and JEV, as well as many other viruses including the alphavirus CHIKV (reviewed in [96,97]). However, clinical and epidemiological data for arbovirus ADE other then DENV is lacking, and thus the clinical significance of these studies remains unknown and requires further investigation.

## 3 Site-directed attenuation strategies

**Fig. 2:**
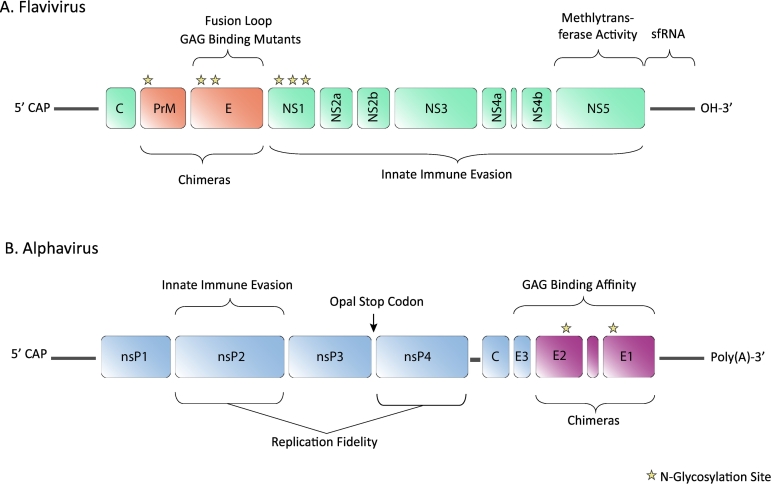
**Flavivirus and alphavirus genome organization and indicated targets for attenuation.** (A) Schematic representation of the flavivirus single-stranded RNA genome. The 10–11 kb genome is flanked by a capped 5’UTR and a highly-structured 3′ untranslated region responsible for the formation of subgenomic-flavivirus RNA (sfRNA). The single open-reading frame codes for one poly-protein which is cleaved into three structural (C, prM, and E) and five non-structural proteins (NS). (B) The alphavirus single-stranded RNA genome of 11–12 kb is capped and polyadenylated, and contains two open-reading frames.The first and second open-reading frame code for four non-structural (nsPs), and structural proteins (C, E1, E2, and E3), respectively. Sites for targeted, attenuating mutations are indicated in the flavivirus and alphavirus genome.

Introducing specific changes to viral genomes that have been described to attenuate virus replication or reduce pathogenicity can help with the rational design of safer and more efficacious LAVs. However, it is unknown how conserved attenuating mechanisms are across related viruses [[98], [99], [100], [101]]. Furthermore, targeted mutations of conserved amino acids do not necessarily result in similar attenuated phenotypes when applied to different virus species or even to different lineages of the same virus [102,103]. However, some promising mechanisms have not only shown consistent attenuation across different viral species within a genus, but were also found to be applicable to both flaviviruses and alphaviruses (Fig. 2 and Table 1).

**Table 1:** Summary of conserved attenuating mutations across multiple A) flaviviruses and B) alphaviruses.

	Target	Outcome Summary	Viruses	Model Used	Ref.
A) Flaviviruses:					
	PrM Glycosylation	Removal of the PrM glycosylation site attenuates neuroinvasion and neurovirulence in mice (JEV) and inhibits viral release and spread *in vitro* (ZIKV).	JEV ZIKV	ICR mice (i.p/i.c) *In vitro*: Vero Cells	[138] [116]
	Env Glycosylation	Removal of the envelope glycosylation site consistently results in increased survival and reduced viral loads in peripheral infection (i.p or s.c). Reduction in neurovirulence was shown for JEV and WNV, as well as survival against a WT virus challenge.	JEV WNV ZIKV MVEV TBEV TMUV	C57BL/6 mice (i.p/i.c) Swiss mice (i.p/i.c) A129 mice (s.c/i.c) Swiss mice (i.p) C57BL/6 mice (s.c) Cherry Valley ducks (i.c)	[120] [114,121] [139] [140] [141] [142]
	GAG binding affinity	Incorporating positive charge amino acids into the envelope which enhance affinity for GAG receptors results in increased survival and reduced viral loads from peripheral infection (i.p or s.c), and an increased rate of viral clearance from blood (i.v). TBEV results also show survival against a WT virus challenge. Neurovirulence (i.c) however is either maintained or increased.	JEV YFV WNV DENV MVEV TBEV TMUV	Swiss Outbred mice (i.v, i.c, i.p) IFN-α/γ-R−/− mice (i.v, s.c) Swiss Outbred mice (i.p, i.c), BALB/c mice (i.v,) IFN-α/γ-R−/− mice (i.p, i.v, i.c) Swiss Outbred mice (i.p, i.c) Swiss Outbred (s.c), ICR mice (s.c, i.c) Pekin ducklings (i.c, s.c)	[106,143] [144] [143] [145] [146] [147] [148] [149]
	ENV Fusion site	L107F mutation is implicated in attenuation for JEV, and was attenuating WNV in peripheral infection (i.p).	JEV WNV	ICR mice (i.c) ICR mice & NIH Swiss mice (i.p, i.c)	[150] [114]
	NS1 Glycosylation	Removal of one or more glycosylation sites results in an attenuated phenotype, but there is variation in which specific sites and whether a peripheral or a neurovirulence model was used.	YFV DENV WNV TMUV	ICR mice (i.c) ICR mice (i.c) NIH Swiss mice (i.c, i.p) Ducklings (i.m)	[151] [152] [153] [149]
	NS5 Methyl-transferase	Disrupting the active site results in attenuation in a peripheral infection model, as well as survival against a WT virus challenge.	JEV WNV	BALB/c mice (i.p, i.c) C3H mice (s.c, i.p)	[154] [123]
	sfRNA	Mutations and deletions that disrupt sfRNA attenuate WNV, ZIKV and DENV.	WNV DENV	Swiss outbred mice (i.p.)	[125,155,156]

^B) Alphaviruses:^					
	Env Glycosylation	Removal of the E1 site is attenuating, but the E2 site had little effect. Removal of the E1 site was less virulent but removal of the E2 site enhanced neurovirulence.	RRV SINV	C57BL/6 mice (s.c) CD-1 mice, C57BL/6 mice (i.c)	[121] [122]
	GAG binding affinity	Incorporating positive charge amino acids into the envelope which enhance affinity for GAG receptors results in increased survival and reduced viral loads from peripheral infection (i.p, s.c) and increased rate of viral clearance from blood (i.v). Neurovirulence (i.c) however is either maintained or increased.	CHIKV SFV SINV VEEV EEEV⁎	CD-1 mice, STAT129 mice (s.c) BALB/c mice (i.p, i.c) Neonatal ICR-L+ mice (s.c) CD-1 mice (s.c, i.v) CD-1 mice (s.c, i.c)	[157] [158] [159] [109] [112]
	nsP2 IFN Signalling	Mutations of conserved proline disrupts nsP2 function in vitro (CHIKV) and attenuates virulence in mice (SINV).	SINV CHIKV	Suckling mice (i.c) *In vitro*: Vero Cells	[122] [124]
	nsP3 Opal Site	Conflicting results (whether removing or adding a stop codon is attenuating is virus dependent).	SFV SINV CHIKV	BALB/c AnNHsd mice (i.p) CD-1 mice (i.c) C57BL/6 J (s.c)	[160] [161] [162]

⁎ Study looked at removal of positive charge amino acid in naturally neurovirulent strain. Intraperitoneal (i.p), Subcutaneous (s.c), Intracranial (i.c), intravenous (i.v),

A promising strategy to attenuate flavivirus and alphavirus entry are targeted mutations in the envelope (E) proteins that enhance glycosaminoglycan (GAG) affinity. GAGs are hydrophilic polysaccharides found in mammalian connective tissues, on cell surfaces and the extracellular matrix, which can act as receptors for a number of viruses. In animal models, multiple flaviviruses and alphaviruses with increased GAG affinity were found to be sequestered in the extracellular matrix and in GAG-rich organs. This resulted in reduced neuroinvasiveness and increased the rate of viral clearance from the blood, which subsequently led to improved survival rates (Table 1) [99,101,[104], [105], [106], [107], [108], [109]]. Furthermore, pre-exposure to an attenuated CHIKV GAG-mutant protected mice upon a subsequent challenge with wild-type virus [110]. Morevover, increased GAG affinity is also considered to be the mode of action for one of the mutations associated with the attenuation of the life-attenuated JEV SA14–14-2 vaccine [111]. However, GAG binding has also been observed to promote infection in specific tissues which highlights the complexity of manipulating viral pathology through GAG-associated mutations alone [107,112]. In addition to GAG affinity, mutations located in the fusion domain of the JEV SA14–14-2 E protein attenuated virus replication [113]. This fusion site is highly conserved among flaviviruses, and introducing a homologous mutation in WNV also resulted in attenuation in mice (Table 1) [114].

Other promising targets are N-glycosylation sites. N-glycosylation sites are highly conserved motifs which are present in both flaviviruses and alphaviruses. N-linked glycosylation is the most common post-translational modification of proteins and it can affect a plethora of processes including protein folding, transport and receptor binding. N-glycosylation of viral proteins can therefore affect virus infectivity for example by modulatiing virus replication, assembly, attachment and cell entry [[115], [116], [117]]. Targeted removal of N-glycosylation sites in either the PrM [116], E or NS1 protein of flaviviruses (reviewed in [117]), or the E1 domain of the alphavirus E protein [118,119], consistently resulted in attenuation in animal models (Table 1). Furthermore, mice pre-exposed to JEV and WNV bearing N-glycosylation site mutations in the E protein were protected upon subsequent challenge with corresponding wild-type viruses [120,121].

In addition to mutations in structural proteins, specific amino acid residues in nonstructural proteins that are involved in innate immune evasion, such as NS5 in flaviviruses, and a conserved proline in nsP2 of alphaviruses are appealing targets for virus attenuation. Mutations in both proteins have shown to attenuate virulence in animal models (Table1) [[122], [123], [124]]. Furthermore, mutations in the flavivirus non-coding regions e.g. subgenomic flavivirus RNA (sfRNA) attenuate DENV and WNV replication *in vitro* and WNV *in vivo* in vertebrates [[125], [126], [127], [128], [129]] and mosquitoes [[130], [131], [132], [133], [134]]. Establishing whether such mutations could be used more broadly will require more comprehensive research [98,99]. An alternative strategy is disrupting replication fidelity of a virus. Although YFV 17D was shown to have increased replication fidelity, it remains to be shown that either increasing or decreasing the fidelity can result in reliable attenuated vaccine candidates [19,135].

It is clear however that multiple mutations contribute to attenuation of JEV SA14–14-2, as well YFV 17D, and this is an important note for rationally designed LAVs in order to minimize the risk of reversion to virulence. Incorporating multiple attenuating mutations and carefully assessing the stability of the designed changes as well as whether compensating mutations evolve will be important for evaluating the safety of any vaccine candidate [136,137].

## 4 Chimeric virus vaccines

Instead of introducing attenuating mutations, the narrow quasispecies diversity and genomic stability of YFV 17D offers opportunities for the use of the YFV 17D genetic backbone for the development of vaccines by chimerization [163]. ChimeriVax (Sanfoni Pasteur) is a vaccine platform designed to swap the structural prM and E proteins of YFV 17D for heterologous flavivirus prME resulting in recombinant LAVs with the expected safety profile of YFV 17D (Fig. 2). Numerous YFV 17D chimeric vaccine candidates have been developed using the ChimeriVax technology including Dengvaxia (DENV1, DENV2. DENV3, and DENV4), IMOJEV (JEV), ChimeriVax-Zika (CYZ; ZIKV) [164] and ChimeriVax-WN02 (WNV) (reviewed in [165]). For all four candidates, low levels of viremia and high titers of neutralizing antibodies were observed in human clinical trials [[164], [165], [166], [167]]. Furthermore, chimeric YFV 17D with ZIKV prM and E was found to protect mice from a lethal challenge of not only ZIKV, but also YFV in mouse model systems [168]. IMOJEV and Dengvaxia are now licenced for human use in Australia and DENV endemic countries, respectively. However, for Dengvaxia low vaccine efficacy against some serotypes and ADE resulting in severe dengue disease was observed in vaccinated seronegative individuals [169,170]. This prominent safety concern mandates serological pre-screening of vaccinees which limits vaccine efficacy and applicability of Dengvaxia, especially in resource-poor settings. Next to Dengvaxia, two other DENV tetravalent chimeric vaccine candidates are currently in development. Takeda's tetravalent chimeric DENV vaccine candidate (TAK-003) is constructed using the backbone of the attenuated DENV serotype-2 PDK-53 with inserted the prM and E genes of the other three DENV serotypes [171]. Unlike Dengvaxia, TAK-003 has shown to be efficacious and safe for use in both seronegative and seropositive participants and is now approved in Brazil [[172], [173], [174], [175]]. The attenuated DENV-2 PDK-53 has also been successfully used to develop chimeric vaccine candidates for ZIKV and WNV that protected animals against a subsequent lethal challenge with ZIKV and WNV [176,177]. Butantan-DV tetravalent vaccine candidate (TV-003/005) is formulated as a mixture of DENV1, DENV3, and DENV4 with a 30 nucleotide deletion in their 3’UTR (DENV1/3/4-Δ30), and chimeric DEN2/4Δ30 with DENV2 prM and E genes inserted in the attenuated DENV4Δ30 backbone [178]. In a clinical phase II trial, vaccination with Butantan-DV was safe and well tolerated, and showed the induction of a well-balanced B-cell and T-cell response against all four DENV serotypes [179]. The induction of a balanced immune response is desirable as it is believed that next to the quality, the quantity of pre-existing antibodies can modulate the host's immune response upon a subsequent challenge or vaccination (reviewed in [180]). The live-attenuated JEV SA14–14-2 has been used as a backbone to develop a ZIKV chimeric vaccine candidate (ChinZIKV). ChinZIKV induces strong and long-lasting immunity and fully protected adult mice and fetus, as well as rhesus macaques against ZIKV challenge [181]. To create alphavirus chimeric vaccines, a relatively non-pathogenic virus like Sindbis virus (SINV) or an attenuated virus strain like VEEV TC-83 or Eastern equine encephalitis (EEEV) BeAr436087 has been used to exchange E1/E2 proteins (Fig. 2). The chimeric vaccine candidates SINV/CHIKV, VEEV/CHIKV, and EEEV /CHIKV showed attenuation, induced high levels of neutralizing antibodies and protected mice upon lethal challenges with CHIKV [[182], [183], [184]]. Also WEEV/EEEV chimera were attenuated and protected mice against a lethal challenge with EEEV [185].

Another promising strategy for making chimeric vaccines is using insect-specific relatives of flaviviruses and alphaviruses. Even though phylogenetic studies indicate that insect-specific viruses (ISVs) are related to vertebrate infecting viruses, empirical studies provide experimental evidence that they are restricted to replication in insects [[186], [187], [188], [189]]. This allows for the design of safe chimeric vaccines using the genetic backbone of ISVs with the structural cassette of pathogenic arboviruses. The insect-specific alphavirus Eilat virus (EILV) was the first ISV used to create a chimeric vaccine candidate with CHIKV. The EILV/CHIKV chimeric virion is structurally identical to wild-type CHIKV and able to enter and deliver its viral RNA in vertebrate cells. However, the chimeric RNA is replication incompetent both *in vitro* and *in vivo* in vertebrates. Using EILV/CHIKV for vaccination showed high and robust levels of immunogenicity and protected mice from subsequent CHIKV infection [190]. Similarly, the insect-specific flavivirus Binjari virus (BinJV) was used to create BinJV/ZIKV, BinJV/WNV, BinJV/DENV-2, and BinJV/YFV chimera for vaccination purposes [[191], [192], [193]]. BinJV chimera were able to enter vertebrate cells, yet failed to produce progeny virus, and showed high levels of protective immunity in mouse models. ISV/arbovirus chimera are still able to grow to high titers in mosquito cells, which can be exploited for the production of these ISV/arbovirus chimeric vaccines.

## 5 Recoded virus vaccines

**Fig. 3:**
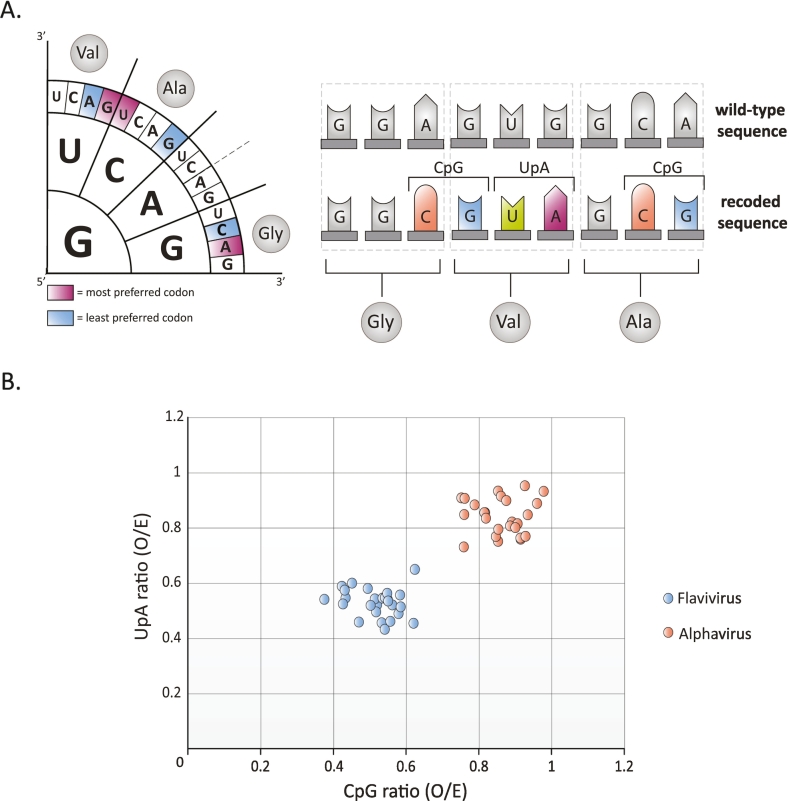
**Codon and dinucleotide usage in flaviviruses and alphaviruses.** (A) Viral genomes can be recoded with synonymous changes that affect the codon usage, codon-pair usage and/or dinucleotide frequencies. (B) Data points represent observed/expected (O/E) CpG (x-axis) and UpA (y-axis) dinucletotide-frequencies, with the expected frequencies calculated from a random distribution of the RNA's mononucleotides. Data points represent full length genomes of distinct vertebrate-infecting flavivirus species (blue) and alphavirus species (orange). (For interpretation of the references to colour in this figure legend, the reader is referred to the web version of this article.)

Eighteen of the twenty amino acids are encoded by more than one codon. This redundancy in the genetic code leaves room for selective use of nucleotides and codons without changing the encoded protein through synonymous mutations. Multiples of such synonymous mutations can be used to recode viral genomes and specifically alter the nucleotide, dinucleotide and codon (pair) usage frequencies. While this strategy results in “silent” changes to the protein product, synonymous recoding can affect the fate of the RNA with potential effects on RNA turnover, translation efficacy, RNA replication, and engagement with cellular factors that recognize certain RNA patterns (reviewed in [194]). This provides opportunities for the design and engineering of LAVs as the viral genome can be intentionally deoptimized for replication in a host by means of synonymous changes (Fig. 3A).

Most viruses display codon usage frequencies that reflect the genome composition of the host. This mimicry is reasonable because a virus uses the host's machinery for the translation of viral proteins, resulting in the optimal use of the host's available tRNAs to recognize the viral codons. Moreover, certain codon pairs are used more frequently, and other pairs are avoided; this is known as codon pair bias. A ribosome decodes codon pairs during translation; thus, codon pair bias alters the translation elongation rate and may alter protein folding and the coordinated expression of functionally grouped proteins [[195], [196], [197]]. Large-scale genomic synonymous recoding such as random codon shuffling, codon-deoptimization, and codon-pair deoptimization all resulted in attenuated replication of CHIKV, DENV, TBEV, and ZIKV in vertebrate and invertebrate cells [[198], [199], [200], [201], [202]]. In addition, synonymously changing codons to be one mutation away from becoming a stop codon decreased the mutational robustness of CHIKV and attenuated virus replication *in vitro* and *in vivo* in mice and mosquitoes [203]. Furthermore, randomly recoded TBEV and codon-pair deoptimized ZIKV showed an attenuated phenotype *in vivo* and protected mice upon subsequent lethal challenges with wild-type virus, and blocked the vertical transmission of ZIKV during pregnancy [200,204]. However, changing the distribution of codons and codon-pairs correspondingly alters the (di)nucleotide usage and recent studies suggest that the avoidance of CpG and UpA is a main driver for the observed codon distribution in viral genomes (Fig. 3A) [[205], [206], [207], [208], [209]].

The suppression of CpG and UpA dinucleotides in viral genomes mirrors the underrepresentation of these dinucleotides in the RNA of humans and other vertebrate animals. Many vertebrate RNA viruses including flaviviruses have evolved to suppress their CpG and UpA dinucleotides (Fig. 3B) [210,211]. The intentional introduction of hundreds of CpG and/or UpA dinucleotides by synonymous recoding attenuates the replication of diverse vertebrate viruses including ZIKV and protected mice upon subsequent challenge with wild-type ZIKV [212,213]. In vertebrate cells, sequences rich in CpG dinucleotides are recognized by the vertebrate zinc-finger antiviral protein (ZAP). ZAP is connected with the IFN response, stimulates RNA degradation and inhibits translation initiation [[214], [215], [216], [217], [218], [219], [220]]. Knockout of ZAP rescues the attenuated phenotype of CpG-high virus mutants and in some studies also improve replication of attenuated UpA-high virus [212,216]. However, in what way ZAP is involved in the attenuation of UpA-high virus mutants is unclear [221].

In contrast to vertebrates, mosquito RNA contains unbiased frequencies of CpG dinucleotides. Interestingly, CpG-high ZIKV mutants that were attenuated in vertebrate cells displayed enhanced replication rates in mosquito cells and improved virus dissemination to the salivary glands in live mosquitoes compared to wild-type ZIKV [212]. This strongly suggests that deoptimizing arboviruses for replication in vertebrate cells can improve virus replication in mosquito cells. Compared to flaviviruses, alphaviruses have evolved higher CpG dinucleotide frequencies in their genomes, which is more similar to the unbiased frequencies in mosquito RNA (Fig. 3B). Wild-type alphaviruses are highly sensitive to overexpression of vertebrate ZAP [222]. However, it remains to be tested whether further elevating CpG dinucleotides in alphaviruses attenuates replication in the vertebrate host.

Synonymously recoded, attenuated viruses have protein structures that are identical to wild-type viruses and are therefore expected to induce a specific and strong immune response. Attenuation by recoding involves the introduction of hundreds nucleotide substitutions that all contribute to the attenuated phenotype, thereby greatly reducing the risk of reversion to wild-type virulence [198]. Elevating levels of CpG and UpA dinucleotides has the potential to attenuate mosquito-borne viruses as well as many vertebrate-specific viruses (reviewed in [194]). Elucidating underlying molecular mechanisms of attenuation provides a rationale for the safe use of CpG-high mutant viruses and opportunities to grow attenuated viruses in specific knockout cells. Moreover, CpG-high LAVs can also grow to high titers in mosquito cells. However, there is little data available on the safety and efficacy of this approach. It will therefore be imperative to investigate the level of attenuation and potential infection of specific tissues (e.g. central nervous system, placenta). With the observed enhanced replication in mosquitoes, also the potential for vaccine transmission and persistent infections in mosquito populations need to be taken into account during vaccine development.

## 6 Mosquito saliva-based vaccine

While taking a bloodmeal, mosquitoes inject saliva into the host's skin [223,224], inoculating a mixture of several dozen proteins and other bioactive molecules that can affect local virus accumulation and further virus dissemination [225]. Importantly, the presence of mosquito saliva at the bite site skews the local immune balance towards a T-helper (Th) 2 response, which is inferior in restricting virus growth compared to Th1-biased responses [31,[226], [227], [228]]. This increases viral load in the skin and in the blood, and accelerated mortality of the host [29,170,225,[229], [230], [231], [232]].

Considering its virus-enhancing properties, mosquito saliva has emerged as a novel target to impede mosquito-borne virus infections. The concept relies on immunization of individuals with crude mosquito saliva or selected, usually immunomodulatory, saliva proteins to prime an immune response against those components. After an infectious mosquito bite, inhibition of bioactive saliva components should prevent skewing of Th1 to Th2 responses resulting in a more favorable Th1-dominated immune response. Indeed, immunizing mice with a high dose of whole salivary gland homogenate from *Culex tarsalis* increased the production of Th1-type cytokines and, after a mosquito-transmitted WNV challenge, resulted in reduced mortality and less virus dissemination to the brain [233]. Similarly, immunization of mice with *Aedes aegypti* salivary proteins AgBR1 [234,235], NeST1 [236], or a combination of those [237] protected against ZIKV disease after an infectious mosquito bite. While immunization with these immunomodulatory proteins was able to protect against disease in mouse models, vaccine strategies using other saliva components (or cocktails hereof) have resulted in enhanced virus replication or aggravated disease [238,239]. These salivary proteins are unsuitable for vaccine development and caution must be taken when creating a vaccine based on individual mosquito salivary proteins.

While some promising proof-of-principle studies for saliva-based vaccine candidates have been reported for pathogens transmitted by sandflies and ticks [[240], [241], [242], [243], [244]], the clinical development of a mosquito saliva-based vaccine is still in its infancy. In a recent phase 1 clinical trial, a single-dose vaccine based on recombinant *Anopheles gambiae* salivary proteins was tested for its safety and immunogenicity in humans [245]. In this study, no safety concerns were identified, while saliva-specific antibody responses and the production of Th1-type cytokines were triggered. Of note, this response was partly dependent on the addition of an adjuvant to the saliva-based vaccine, thus a fraction of the induced immune response was stimulated by the adjuvant rather than the mosquito salivary proteins [246]. The saliva-specific antibodies were maintained for at least 3 months but diminished after 1 year [245]. To date, this is the only study that has evaluated the effect of a mosquito saliva-based vaccine in humans, and although it provides data regarding the immunogenicity of such a vaccine, no pathogen exposure was conducted. Whether it provides humans with protection against pathogens transmitted by *Anopheles gambiae* thus remains to be determined.

Besides the longevity of immune responses, the development of saliva-based vaccines faces additional challenges that need to be addressed: For locals, long-term exposure to mosquito allergens may lead to desensitization, apparent by waning immediate and delayed immune responses to mosquito saliva [247]. This could make a saliva-based vaccine more relevant for naïve travellers who plan short-term visits to endemic areas. Moreover, in a longitudinal study, children with higher antibody levels against *Aedes aegypti* salivary proteins were 1.5 times more likely to develop inapparent DENV infection, challenging the idea of a monovalent saliva-based vaccine strategy [248]. Instead, immunization with salivary proteins could be moved forward as an adjuvant for pathogen-targeted vaccines, a concept that has been tested for sandfly saliva and *Leishmaniasis* infection in mice [249]. In conclusion, while saliva-based vaccines have emerged as new strategy with the potential to affect replication of multiple viruses transmitted by the same mosquito species, many practical hurdles still need to be taken in to account, in particular the identification and validation of effective vaccine targets within the complex blend of mosquito saliva proteins.

## 7 *In vitro* models

Thorough safety assessment of candidate LAVs must be carried out to ensure that pathogenesis and transmission have been sufficiently attenuated, whilst maintaining immunogenicity. As many arboviruses are able to cause severe neurological disease in a subset of infected individuals, *in vitro* models of neuroinvasion and neuropathogenesis can be used to gain insight into the attenuation of these key stages of disease progression.

Such models range in complexity from 2D cell line monocultures to 3D organ/vessel on a chip and *ex vivo* organoids [250,251]. Due to the broad cell tropism of many arboviruses, 2D and 3D co-culture models with relevant neuronal and neurovascular cell types would provide the most complete picture into the degree of attenuation of neuroinvasion and pathogenesis of vaccine candidates. To model the effect of attenuation on virus transmission, *in vitro* and *ex vivo* human skin models and mosquito cell lines can provide a rapid initial indication, but this complex phenotype eventually requires validation in *in vivo* transmission models [252].

To predict immunogenicity and antigenicity of vaccine candidates, *in silico* techniques can be applied [251,253]. This can streamline the vaccine candidate selection process prior to *in vitro* assessment of immunogenicity using primary human, or human derived immune cells to identify cytokine responses and induction of cell maturation and proliferation [253]. For some viruses the route of vaccination may influence the subsequent immune response [254,255], so more complex 3D models that recapitulate features of the human immune system within relevant physiological contexts, such as skin, could also be employed to refine assessment of immunogenicity and act as a bridge to successive *in vivo* studies.

Indeed, whilst *in silico* and *in vitro* models are essential tools in the development and selection of safe and effective arboviral vaccine candidates, the data obtained cannot be fully extrapolated to the physiological setting. Further, characteristics such as the viraemic profile and transmissibility of LAV candidates cannot be obtained *in vitro*.

## 8 *In vivo* models to assess vaccine safety and efficacy

Assessing vaccine efficacy *in vivo* requires challenging vaccinated animals with virus. This is generally done via needle-inoculation, typically via the intraperitoneal route. Importantly, needle-delivery of arboviruses does not model important parameters of the natural infection, such as immunomodulatory factors of mosquito saliva that are co-inoculated into the bite site during virus transmission [224]. These salivary factors play a key role in the establishment and potentiation of virus infection in the vertebrate host [31,[226], [227], [228]]. When hamsters were vaccinated against VEEV they were fully protected from both needle- and infectious mosquito-challenge [256]. Likewise, challenging mice [257], cats and dogs [258], or horses [257,259] via an infectious mosquito bite subsequent to WNV vaccination resulted in protection against WNV disease and no development of detectable viremia. However, comparing needle-inoculation with mosquito-delivery of arboviruses in animal models shows differential immune responses [227], viremia [[260], [261], [262], [263]], disease progression [225,262], and tissue tropism [261]. These aspects should be taken into account when assessing vaccine efficacy and safety *in vivo*, considering arboviruses and other arthropod-borne pathogens can become more virulent when inoculated via a mosquito bite [29]. For example, when *Plasmodium*-vaccinated mice were challenged with *Plasmodium* sporozoite infection, the needle-challenged mice were fully protected whereas protection was significantly limited for mice challenged via a mosquito bite [264]. Comparably, vaccination against *Leishmania* protected mice against subsequent needle-challenge with *Leishmania*, but this protection was completely abolished when mice were challenged via infected sandfly exposure [265]. These findings highlight the fact that conventional *in vivo* challenge models cannot be readily extrapolated to the natural setting. Even so, vaccination studies employing a mosquito-challenge mouse model to predict the protective efficacy of arbovirus vaccines in humans are not typically implemented.

For *in vivo* testing of vaccine candidates, employing (i) infectious mosquito biting, (ii) non-infectious mosquito probing prior to challenge, or (iii) co-inoculation of virus with mosquito saliva or salivary gland extract as standard for challenge may aid in predicting vaccination efficacy against natural exposure to infectious mosquitoes. Furthermore, *in vivo* models can aid in testing the likelihood of LAVs to be taken up from an inoculated animal by vector mosquitoes and thus the transmission potential of LAVs.

## 9 Future perspectives

Classical LAVs, like YFV 17D, have been shown to be more successful in disease prevention than their inactivated counterparts. LAVs are similar to natural infection in their antigen load and presentation to the immune system. Therefore, LAVs are strong inducers of the innate and adaptive response resulting in generation of strain specific T-cells and neutralizing antibodies that confer (life-long) protection. Classical LAVs (e.g. YFV 17D, vaccinia against smallpox, measles/mumps/rubella vaccine, oral poliovirus vaccine) have been safely used in humans for decades. Nevertheless, attenuation by repeated passage is the result of random mutations [266], and poses several risks (e.g. reversion to wild-type and pathogenicity in immunocompromised individuals) that limit the development of new LAVs [267]. Therefore, novel approaches need to be safe-by-design and provide the desired level of attenuation and sufficient stability of the attenuating mutations. Combining multiple mutations in conserved functional sites, generating chimeras with established, attenuated viral backbones or genome-wide recoding are promising strategies to design novel LAV candidates. These attenuating strategies can also be combined to further reduce replication and/or pathogenicity, e.g. mutations in specific neurotropic-related residues in the E protein of YFV/WNV and YFV/JEV chimeras [113,268]. In addition to creating novel LAVs, mosquito salivary proteins may be utilized as pan arbovirus vaccines or adjuvants to enhance protection against arbovirus disease.

Preclinical evaluation of novel arbovirus LAV candidates requires relevant *in vitro* and *in vivo* model systems to assess pathogenicity in different tissues and the quality of immune responses that are induced. Moreover, a natural viral infection via mosquito bite can change the outcome of infection compared to challenge by needle injection. It is therefore crucial that novel mosquito-borne virus vaccines are also evaluated in the context of a natural transmission route. Direct comparisons of novel LAVs with established vaccines such as YFV 17D may provide valuable information on e.g. the functionality, breadth and longevity of elicited T and B-cell responses. It is important to note that although we discuss broadly applicable vaccination strategies, there are substantial differences in tissue tropism, pathogenicity and outcome of disease between viral species that need to be taken into account during (pre-)clinical evaluations. The prime example is ADE of dengue virus infections. The tetravalent chimeric vaccine Dengvaxia, successfully passed all (pre-)clinical evaluations and vaccination resulted in high and robust levels of neutralizing antibodies. However, upon implementation, ADE resulting in severe dengue disease was observed in vaccinated seronegative individuals [169,170]. Although there is currently no clinical and epidemiological data that describes ADE of other arbovirus infections, experimental evidence from multiple arbovirus species warrants the careful consideration of potential negative immune interactions between different arboviruses [95,96].

The combination of broadly applicable vaccination strategies and relevant *in vitro* and *in vivo* model systems to assess vaccine safety and efficacy will aid in the rapid development of novel arbovirus vaccines. Particularly, mosquito transmission and challenge experiments can provide additional data on the efficacy of vaccines against natural infections. Together, the topics discussed can provide promising strategies to target the direct interface of the vertebrate host, the mosquito vector and the viral pathogen, with potential to alleviate arbovirus disease burden in human and animal populations.

## CRediT authorship contribution statement

**Joyce W.M. van Bree:** Investigation, Visualization, Writing – original draft, Writing – review & editing. **Imke Visser:** Investigation, Writing – original draft. **Jo M. Duyvestyn:** Investigation, Writing – original draft. **Muriel Aguilar-Bretones:** Investigation, Writing – original draft. **Eleanor M. Marshall:** Investigation, Writing – original draft. **Martijn J. van Hemert:** Writing – review & editing, Supervision. **Gorben P. Pijlman:** Writing – review & editing, Supervision. **Gijsbert P. van Nierop:** Writing – review & editing, Supervision. **Marjolein Kikkert:** Writing – review & editing, Supervision. **Barry H.G. Rockx:** Writing – review & editing, Supervision. **Pascal Miesen:** Investigation, Writing – original draft, Writing – review & editing. **Jelke J. Fros:** Investigation, Writing – review & editing, Supervision.

## Declaration of Competing Interest

The authors declare that they have no known competing financial interests or personal relationships that could have appeared to influence the work reported in this paper.

## Data Availability

No data was used for the research described in the article.
